# Factors associated with adherence to the Mediterranean diet in patients with coronary artery disease: a prospective cohort study

**DOI:** 10.1590/0034-7167-2025-0248

**Published:** 2026-08-03

**Authors:** Melissa Alves Cirelli, Juliana de Lima Lopes, Aline Marcadenti, Vinícius Batista Santos, Agueda Maria Ruiz Zimmer Cavalcante, Alba Lucia Bottura Leite de Barros

**Affiliations:** I Universidade Federal de São Paulo. São Paulo, São Paulo, Brazil; II Instituto de Ensino e Pesquisa Hcor. São Paulo, São Paulo, Brazil; III Universidade Federal de Goiás. Goiânia, Goiás, Brazil

**Keywords:** Diet, Mediterranean, Coronary Artery Disease, Sociodemographic Factors, Nursing, Cohort Studies., Dieta Mediterránea, Enfermedad de la Arteria Coronaria, Factores Sociodemográficos, Enfermería, Estudios de Cohortes.

## Abstract

**Objectives:**

to verify the association between adherence to the Mediterranean diet (MedDiet), sociodemographic, clinical, and biochemical factors, and hospital readmission in patients with coronary artery disease.

**Methods:**

prospective cohort study in a tertiary hospital. MedDiet adherence was analyzed at admission and at three and six months after discharge using the Mediterranean Diet Adherence Screener. C-reactive protein, total cholesterol, LDL cholesterol, and triglycerides were analyzed at admission and six months after discharge; readmission was assessed at three and six months. Associations were verified using the generalized estimating equation model, with p-values ≤0.05 considered significant.

**Results:**

a total of 146 patients participated, mostly men, with a mean age of 66 years. MedDiet adherence increased from 3% to 41% and 47% during follow-up and was associated with age, female gender, complete higher education, economic activity, and previous myocardial revascularization.

**Conclusions:**

MedDiet adherence was associated with sociodemographic and clinical factors.

## RESUMEN

**Objetivos:**

verificar la asociación entre la adherencia a la dieta mediterránea (MedDiet), factores sociodemográficos, clínicos y bioquímicos y la readmisión hospitalaria en pacientes con enfermedad arterial coronaria.

**Métodos:**

cohorte prospectiva en un hospital terciario. La adherencia a la MedDiet se analizó al ingreso y en tres y seis meses del alta mediante el *Mediterranean Diet Adherence Screener.* Se analizaron la proteína C reactiva, el colesterol total, el LDL y los triglicéridos al ingreso y seis meses después del alta; y la readmisión a los tres y seis meses. La asociación se verificó mediante ecuaciones de estimación generalizadas (p≤0,05).

**Resultados:**

participaron 146 pacientes, la mayoría hombres, edad media de 66 años. La adherencia aumentó del 3% al 41% y 47% durante el seguimiento y se asoció con edad, sexo femenino, educación superior completa, actividad económica y revascularización previa.

**Conclusiones:**

la adherencia a la MedDiet se asoció con factores sociodemográficos y clínicos.

## INTRODUCTION

Cardiovascular diseases (CVDs) are a global public health issue, whose growing prevalence has a financial impact and compromises individuals’ productivity and quality of life^(1)^. According to updated data from the American Heart Association (2025), CVDs remain the leading cause of death worldwide, with a growing trend in developing countries^(1)^.

In Brazil, CVDs are the leading cause of death, with coronary artery disease (CAD) accounting for the highest proportion of cardiovascular deaths, followed by stroke. The economic impact is considerable, with annual costs to the Brazilian Unified Health System (SUS) exceeding R$ 1 billion in cardiovascular procedures^(2)^. Mortality from CAD is inversely associated with socioeconomic status, a striking feature of developing countries, aggravated by modifiable risk factors such as obesity, smoking, physical inactivity, and poor dietary habits^(2)^.

The pathophysiology of CAD involves complex atherogenic mechanisms, in which risk factors such as dyslipidemia (DLP), systemic arterial hypertension (SAH), and smoking play a key role in the progression of cardiovascular disease. These factors promote endothelial dysfunction and increase the permeability of the intima to plasma lipoproteins, especially low-density lipoproteins (LDL)^(3)^. This process triggers a chronic inflammatory response that can result in atherosclerotic plaque instability, rupture with activation of the coagulation cascade, and, consequently, acute arterial occlusion^(4)^.

In this context, the Mediterranean diet (MedDiet)^(3)^ has emerged as a prominent nutritional strategy, widely recommended by international guidelines and consensus for the primary and secondary prevention of CVDs^(4-6)^. Studies show that the MedDiet has anti-inflammatory and antioxidant effects, as evidenced by reduced inflammatory biomarkers and improved endothelial function, providing significant cardiovascular protection. High-impact randomized clinical trials, such as the CORDIOPREV^(7)^ and the Lyon Heart Study^(8)^, have demonstrated the effectiveness of the MedDiet in the secondary prevention of CVDs, with substantial reductions in major cardiovascular events.

However, several sociodemographic, clinical, and biochemical factors can influence adherence to the MedDiet, especially in non-Mediterranean populations. Although the global literature strongly supports the benefits of the MedDiet, studies examining the factors that determine adherence to this dietary pattern in secondary cardiovascular prevention within Latin American countries are limited. This gap is especially important given the cultural, socioeconomic, and food availability differences that may affect the adoption and maintenance of this dietary pattern in Brazilian populations with established CAD.

## OBJECTIVES

Identify the association between adherence to the MedDiet and sociodemographic, clinical, and biochemical factors, and hospital readmission in patients with coronary artery disease.

## METHODS

### Ethical aspects

The study was submitted to and approved by the research ethics committees of the institutions involved, in accordance with Resolution No. 466/2012 of the National Health Council.

### Study design

This is a prospective cohort study, guided by the STROBE tool^(9)^, and conducted at a large, private, tertiary general hospital in São Paulo. The study was conducted in two care units: the Cardiac Critical Care Unit (CCU), with 45 semi-critical care beds, a monthly average of 56 admissions per CAD, an average length of stay of three days, and an occupancy rate of 95%; and the Inpatient Unit (IU), with 31 low-complexity beds for circulatory conditions, an average length of stay of three days, and an occupancy rate of 81%.

### Sample calculation and collection period

Data collection took place between July 2021 and July 2022. The sample calculation was based on a previous study^(10)^, which reported a prevalence of 25.4% for Mediterranean diet adherence in a population of 18,991 individuals aged 35 or older. For an infinite sample, an 80% confidence interval, and a sampling error of 5%, the minimum sample size was 125 patients. Anticipating a 20% loss rate, a sample of 149 patients was selected, yielding a final sample of 146 patients diagnosed with CAD.

The formula used was: n = [EDFF*Np(1-p)]/ [(d2/Z21-α/2*(N-1)+p*(1-p)].

### Eligibility criteria

Patients over 18 years of age hospitalized for coronary artery disease (CAD), classified according to established clinical guidelines, were included:

1. Acute CAD: Defined by the presence of acute coronary syndrome, diagnosed using clinical, electrocardiographic, and laboratory criteria, including:
  - Unstable angina (chest pain at rest with transient electrocardiographic changes);
  - Acute myocardial infarction with ST-segment elevation (elevation ≥1 mm in two contiguous leads); - Acute myocardial infarction without ST-segment elevation (ST-segment depression ≥0.5 mm and/or T wave inversion, associated with elevated cardiac biomarkers).
2. Chronic CAD: Characterized by stable coronary artery disease, diagnosed by noninvasive methods (treadmill test, stress echocardiogram, myocardial scintigraphy) or invasive methods (coronary angiography), including:
  - Stable angina (chest pain on exertion, with a predictable pattern);
  - Exertional dyspnea related to myocardial ischemia;
  - Heart failure of ischemic etiology or left ventricular dysfunction; - Asymptomatic patients or those who have had stable symptoms for less than one year after an acute coronary syndrome; - Patients who have undergone myocardial revascularization (percutaneous or surgical) less than one year ago; - Patients with stable symptoms for more than one year after an initial diagnosis or revascularization; - Suspected vasospastic or microvascular disease with positive functional testing.
3. CAD detected in screening of asymptomatic patients.

The following cases were excluded:

1. Cancer patients undergoing active chemotherapy: Given the need for specific dietary prescriptions for this population, which could interfere with the assessment of adherence to the Mediterranean diet.
2. Cognitive or neurological impairment: Patients with mental confusion, decreased level of consciousness, or under sedation, assessed daily by the medical and nursing teams through:
  - Glasgow Coma Scale <15 points;
  - Clinical assessment documented in medical records regarding the state of consciousness;
  - Use of sedatives that compromise the ability to respond.
3. Food allergies: Individuals with allergies documented in medical records to components of the Mediterranean diet (nuts, fish, olive oil, among others), according to medical records.
4. Impossibility of contact: Patients who did not respond to telephone contact after three attempts at different times were considered sample loss, not exclusion criteria, according to the STROBE checklist recommendations for observational studies.

### Protocol and study period

Recruitment: daily identification by the researcher through active search for patients with CAD and hospital discharge scheduling. Patients were approached in person at the inpatient unit and invited to participate after receiving an explanation of the study procedures.

Data collection instrument: structured questionnaire covering sociodemographic and clinical variables, selected through a narrative review of the Medline, Lilacs, and Cochrane databases (articles from the last five years, in Portuguese/English/Spanish). Information was obtained directly from patients and supplemented by medical records.

Variables collected: sociodemographic - gender, age, race, marital status, education level, occupation; clinical - length of hospital stay, type of CAD, body mass index (BMI), comorbidities (depression, hypertension, diabetes, dyslipidemia); and laboratory - total cholesterol, LDL cholesterol, triglycerides, and C-reactive protein (CRP)^(11-14)^. The interviews lasted an average of 30 minutes per participant.

The primary outcome was analyzed using the Mediterranean Diet Adherence Screener (MEDAS), an instrument translated and adapted into Brazilian Portuguese^(15)^ comprising 14 questions, each scored from 0 to 1. A score ≥ 10 indicates greater adherence to the MedDiet. The instrument was applied at three different times: in person, up to 48 hours before hospital discharge; and in follow-up by telephone at three and six months after hospital discharge.

Secondary outcomes were analyzed based on CRP, total cholesterol, LDL cholesterol, and triglyceride values obtained during hospitalization and six months after discharge, using data from medical records and/or telephone interviews. Biomarkers were collected during hospitalization by the nursing team in accordance with the following institutional protocols: tourniquet 7.5-10 cm from the puncture site, antisepsis with 70% alcohol in spiral movements, venipuncture at a 30º-45º angle, and storage in specific tubes for analysis at the Fleury® laboratory.

Cholesterol, triglyceride, and LDL cholesterol values are in accordance with the 2017 update of the Brazilian Guidelines on Dyslipidemia and Atherosclerosis Prevention from the Brazilian Society of Cardiology^(3)^. CRP was determined by immunoturbidimetry, and the reference values for cardiovascular risk classification are: below 0.1 mg/dL: low risk; 0.1 to 0.3 mg/dL: intermediate risk; and above 0.3 mg/dL: increased risk. Regarding total cholesterol, the desirable reference value, with or without 12-hour fasting, for age greater than or equal to 20 years, is: less than 190 mg/dL. For triglycerides, the desirable reference value for individuals aged 20 years or older with 12-hour fasting is less than 150 mg/dL. As regards LDL cholesterol, the reference value for age greater than 20 years, with or without fasting for 12 hours, is established for primary assessment of the lipid profile as: optimal: less than 100 mg/dL; desirable: 100 to 129 mg/dL; borderline: 130 to 159 mg/dL; high: 160 to 189 mg/dL; very high: greater than or equal to 190 mg/dL. Hospital readmission was detected through the participant’s report during telephone contact, three and six months after hospital discharge.

### Analysis of results

Qualitative variables were described by absolute and relative frequency; quantitative variables were described by means of central tendency and dispersion. Adherence to MEDAS was estimated with a 95% confidence interval. Generalized Estimating Equation (GEE) models evaluated factors associated with adherence, estimating relative risk using a multiple model.

For missing data >5%, multiple imputation (MICE - Multiple Imputation With Chained Equations) was used. Variables with more than 50% of missing data were excluded from the analyses. The Wilcoxon test compared distributions of quantitative variables. Mixed Generalized Linear Models (MLGM) were used to evaluate biomarker evolution, with likelihood-ratio tests for interaction terms. The level of significance adopted was p<0.05.

## RESULTS

**Figure 1:**
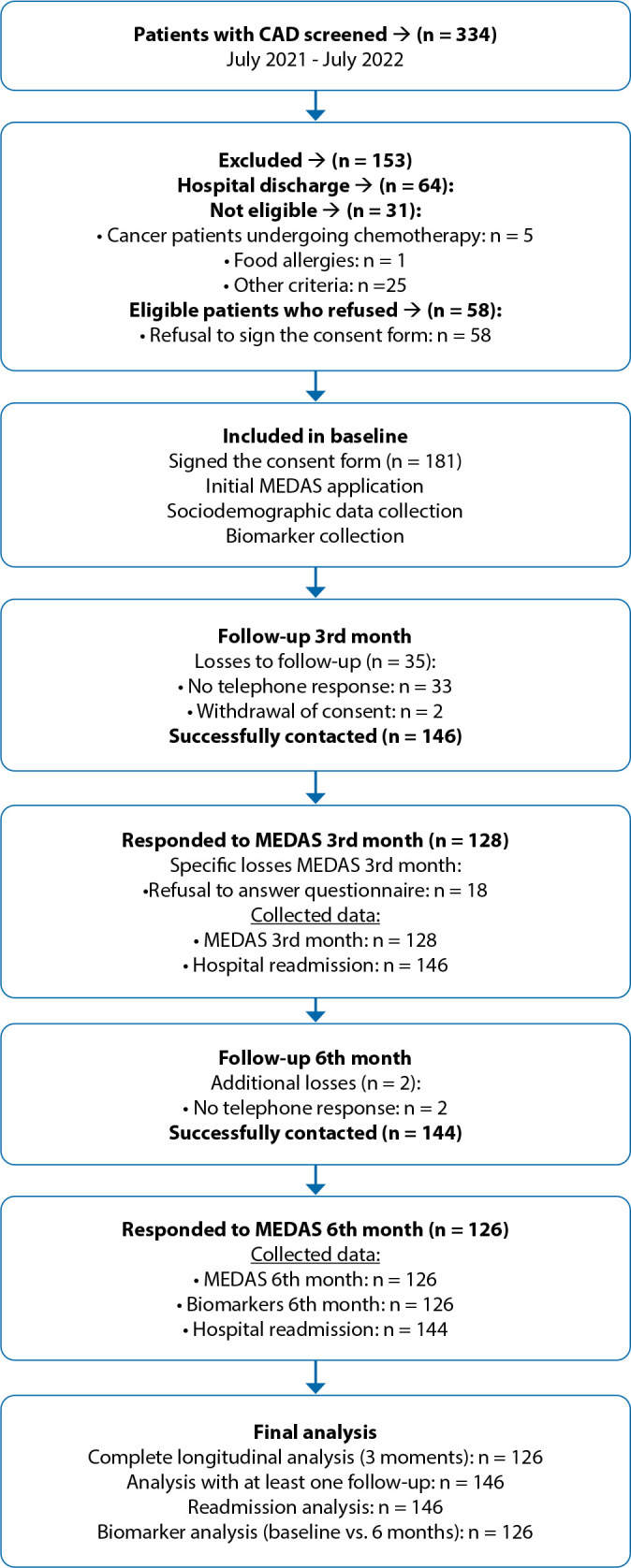
Flowchart of participant selection and follow-up according to the STROBE guidelines, São Paulo, São Paulo, Brazil, 2021-2022

A total of 334 patients with CAD were screened during the collection period. Of these, 181 met the eligibility criteria and agreed to participate in the study by signing the informed consent form. During follow-up, the following losses occurred: 33 patients did not respond to any telephone contact (third and sixth months); 18 did not respond specifically to MEDAS in the third month; two did not respond to contact in the sixth month; and two requested withdrawal from the study. The final sample analyzed comprised 146 patients who responded to at least one telephone contact, of whom 126 completed the entire follow-up (third and sixth months), as shown in Figure 1.

Table 1 shows the sociodemographic and clinical characteristics of the 146 participants. Regarding biomarkers, most patients had CRP levels indicative of high cardiovascular risk (>0.3 mg/dL), followed by intermediate risk (0.1-0.3 mg/dL). Thirteen patients did not have CRP collected during hospitalization. Regarding lipid profile, among patients whose samples were collected during hospitalization, most had total cholesterol and triglyceride values within the desirable range according to Brazilian guidelines.

**Table 1:** Sociodemographic and clinical characteristics and association with adherence to the Mediterranean diet over time in patients with Coronary Artery Disease (N=146), São Paulo, São Paulo, Brazil

Variable	N = 146^*^	RR†	IC95%	*p* value
Length of stay (days)				
Mean (SD)	6.70 (11.56)	0.992	0.976-1.008	0.321
Age				
Mean (SD)	66.27 (12.09)	0.984	0.969-0.999	0.042
Sexo				
Male	117 (80.14%)	0.566	0.350-0.917	0.021
Female	29 (19.86%)	Reference		
Education				
Higher education	126 (86.30%)	Reference		
High school	16 (10.96%)	0.939	0.484-1.825	0.854
Elementary School	4 (2.74%)	0.410	0.183-0.917	0.030
Marital status				
Lives with partner	131 (89.73%)			
Without a partner	15 (10.27%)	0.835	0.398-1.753	0.634
Race				
White	143 (97.95%)			
Not white	3 (2.05%)			
Occupation classification				
Economically active	123 (84.25%)	Reference		
Not economically active	23 (15.75%)	0.377	0.149-0.953	0.039
Medical diagnosis				
Chronic DAC	93 (63.70%)	1.236	0.810-1.888	0.326
Acute DAC	53 (36.30%)			
Personal background				
Sedentarism	111 (76.03%)	Reference		
Hypercholesterolemia	110 (75.34%)	1.037	0.637-1.689	0.883
Systemic arterial hypertension	102 (69.86%)	1.754	1.155-2.662	0.008
Treatment performed				
Angioplasty	115 (78.77%)	Reference		
Clinical	18 (12.33%)	0.935	0.507-1.727	0.831
Surgical	13 (8.90%)	1.614	1.018-2.558	0.042
BMI				
Mean (SD)	27.51 (3.97)	0.985	0.942-1.031	0.517
Maximum/Minimum	17.48. 42.39			
BMI classification				
Overweight	81 (55.48%)			
Regular	33 (22.60%)			
Obese	31 (21.23%)			
Low weight	1 (0.68%)			
CRP				
Mean (SD)	0.65 (1.18)			
Maximum/Minimum	0.03. 7.67			
Absence of results	13			
Cardiovascular Risk Indicator according to the CRP				
High (above 0.3mg /dL)	52 (39.10%)			
Low (below 0.1 mg/dL)	35 (26.32%)			
Intermediate (0.1mg/dL to 0.3mg/dL)	46 (34.59%)			
Absence of results	13			
Total cholesterol				
Mean (SD)	167.09 (51.94)			
Desirable cholesterol <190				
Yes	72 (71.29%)			
LDL				
Mean (SD)	96.36 (42.80)			
Desirable LDL<100				
Yes	64 (63.37%)			
Triglycerides				
Mean (SD)	152.87 (106.01)			
Desirable triglycerides < 150				
Yes	68 (67.33%)			
Prescription drugs				
Antiplatelet agents	113 (77.40%)	1.053	0.685-1.618	0.814
Lipid-lowering agents	103 (70.55%)			
Beta-blockers	56 (38.36%)	1.077	0.698-1.660	0.738

IRR† - Incidence of Relative Risk;

* N (%); SD - Standard Deviation; IC - Interval of Confidence; BMI - Body Mass Index; CAD - Coronary Artery Disease; CRP - C-reactive Protein; SAH - Systemic Arterial Hypertension; DM - Diabetes Mellitus.

In the bivariate analysis, the variables that showed a statistically significant association with greater adherence to MEDAS were: younger age, female gender, higher education, being economically active, presence of hypertension, and having undergone surgical treatment for CAD.

**Figure 2:**
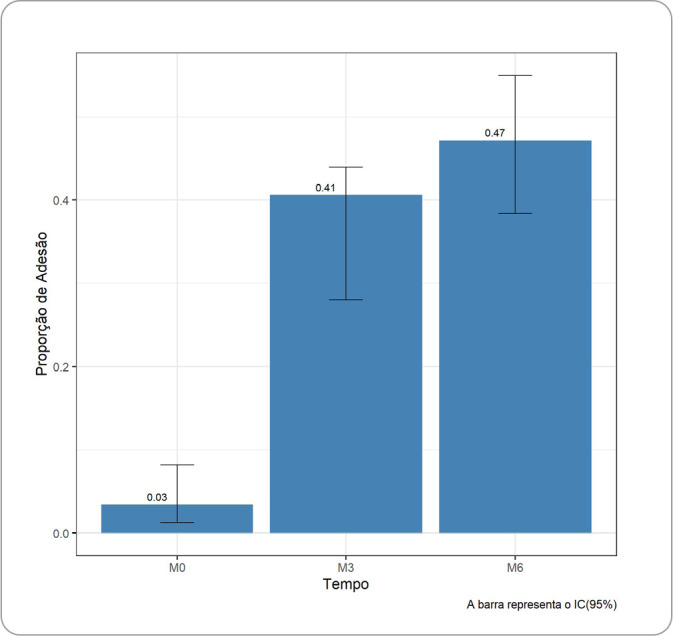
Proportion of adherence to the Mediterranean diet during follow-up of patients with Coronary Artery Disease N=146, São Paulo, São Paulo, Brazil

Figure 2 shows the proportion of participants adhering to the MedDiet (MEDAS≥10) at the three assessment points. A progressive increase in adherence was observed throughout the follow-up period: at hospitalization (baseline), at three months, and at six months after hospital discharge. This finding indicates an improvement in adherence to the Mediterranean diet during the follow-up period.

Table 2 shows the relationship between adherence to the MedDiet and hospital readmission three and six months after discharge. No statistically significant difference was identified between the groups with higher and lower adherence to the diet in terms of readmission rates (p>0.05). Readmission rates remained low throughout the follow-up period.

**Table 2:** Relationship between adherence to the Mediterranean diet during hospitalization and hospital readmission three and six months after discharge, São Paulo, São Paulo, Brazil, 2025

Variable	Hospital readmission (T0)			*p* value^§^
	Total N = 146‡	No n = 143‡	Yes n = 3‡	
**Adherence to the MEDAS T0**				0.101
No	141 (96.55%)	139 (97.18%)	2 (66.67%)	
Yes	5 (3.45%)	4 (2.82%)	1 (33.33%)	
	**Hospital readmission (T6)**			
	**Total N = 144‡**	**No n = 135‡**	**Yes n = 9‡**	
**Adherence to the MEDAS T3**				0.145
No	76 (60.32%)	69 (58.47%)	7 (87.50%)	
Yes	50 (39.68%)	49 (41.53%)	1 (12.50%)	
Absence of results	18	17	1	

*‡n (%); §Fisher’s exact test; MEDAS - Mediterranean Diet Adherence Screener; T0 - time at admission; T6 - time six months after hospital discharge.*

As shown in Table 3, adherence to the MedDiet did not show a statistically significant association with biomarker values at six months of follow-up. However, a significant reduction in total cholesterol and LDL cholesterol levels was observed over time in the entire sample (p<0.05), regardless of adherence to the Mediterranean diet. The analysis did not identify any influence of the interaction between dietary adherence and the use of lipid-lowering drugs on the evolution of biomarkers.

**Table 3:** Association of CRP, total cholesterol, LDL cholesterol, and triglyceride values with adherence to the Mediterranean diet at six months of follow-up in patients with Coronary Artery Disease, São Paulo, São Paulo, Brazil

Covariate	Generalized Linear Mixed Model (GLMM)											
	Total cholesterol			Triglycerides			LDL cholesterol			CRP		
	Beta	IC 95%	*p* value	Beta	IC 95%	*p* value	Beta	IC 95%	*p* value	Beta	IC 95%	*p* value
**Tempo**												
T0	-	-	-	-	-	-	-	-	-	-	-	-
T6	-32.49	-46.5 -18.3	<0.001	0.812	0.69 - 0.95	0.012	0.76	0.64 - 0.89	<0.001	0.75	-0.09 - 1.60	0.082
**Adesão ao MEDAS**												
Não	-	-	-	-	-	-	-	-	-	-	-	-
Sim	-1.86	-21.3 - 1.02	0.841	0.850	0.67 - 1.06	0.162	0.95	0.75 - 1.20	0.701	0.49	-0.69 - 1.68	0.413
**Hipolipomiante**												
Não	-	-	-	-	-	-	-	-	-	-	-	-
Sim	4.32	-34.07 -25.38	0.776	0.901	0.62 - 1.30	0.578	0.896	0.62 - 1.29	0.558	0.34	-0.93 - 1.63	0.594

*GLMM - Generalized Linear Mixed Model; MEDAS - Mediterranean Diet Adherence Screener; T0T6 - time of admission and six months after discharge; M0 - MEDAS admission; M6 - MEDAS six months.*

## DISCUSSION

The results show that adherence to the Mediterranean diet increased progressively over the six-month follow-up period and was associated with younger age, female gender, higher education, economic activity, SAH, and myocardial revascularization. No relationship was observed between adherence to the MedDiet and hospital readmission or inflammatory biomarkers; however, there was a significant reduction in total cholesterol and LDL cholesterol during the follow-up period.

The sociodemographic characteristics of the present study are consistent with those of other cardiovascular cohorts regarding the predominance of males, the average age, and the white race^(16-19)^. The higher prevalence of men reflects epidemiological data on CAD^(1-3,19-21)^ associated with men’s lower adherence to preventive care and delayed seeking of health services^(21)^. The association between female gender and greater adherence to the MedDiet corroborates international findings^(22-26)^, possibly related to the fact that women tend to adopt healthier eating habits, consume more fruits and vegetables, and drink less alcohol than men^(22,26,27)^.

Contrary to trends described in national and international literature, which associate greater adherence to the MedDiet with older age groups^(16,22,25,27)^, this study found that younger individuals showed greater adherence to the Mediterranean diet. This result may be related to the impact of hospitalization on younger patients, who may be more motivated to change their behavior after an acute cardiovascular event, including adopting cardioprotective eating habits, quitting smoking, and starting regular physical activity. Additionally, the pandemic context may have influenced this finding, since multicenter studies conducted during the COVID-19 pandemic demonstrated significant changes in dietary patterns, with increased consumption of fruits and vegetables among young adults, especially in Brazil^(28-31)^. Greater awareness of health and immunity during this period, coupled with greater access to nutrition information on digital platforms, may have contributed to greater adherence to healthy eating patterns among this age group.

The association between higher education levels, being economically active, and greater adherence to the MedDiet confirms the findings in the literature^(25,32)^. Socioeconomic status is a key determinant of access^(2)^ to healthy behaviors and appropriate food choices^(22,33)^. Education directly influences the control of cardiovascular risk factors and adherence to healthy habits, since an increase in socioeconomic status is associated with greater health awareness and better conditions for purchasing quality food^(2,19,21,23,32-35)^. In the Brazilian context, inequalities are exacerbated by political and economic instability, which promotes disparities in access to adequate food in terms of quality and quantity^(28-31)^.

The significant association between SAH and greater adherence to the MedDiet may be related to specialized professional monitoring and greater access to health care, both of which favor behavioral changes. A previous study demonstrated greater adherence to the MedDiet in individuals with established chronic diseases, such as SAH, DM, and DLP^(36)^, possibly due to greater awareness of the importance of diet in controlling these conditions.

The association observed between adherence to the MedDiet and surgical revascularization is an innovative finding that requires further investigation. Although angioplasty is the procedure of choice (58.9%) compared to surgical revascularization (20.1%)^(2)^, patients undergoing cardiac surgery experience a more complex and prolonged hospital stay. This association can be explained by the psychological impact of cardiac surgery, which represents a significant milestone in the perception of disease severity, promoting greater motivation for behavioral changes^(37)^. In addition, surgical patients receive more structured guidance on lifestyle changes during prolonged hospitalization, and the post-surgical rehabilitation process involves a multidisciplinary team, including nursing professionals specialized in nutritional education.

The low number of readmissions observed should be interpreted in light of the pandemic context, which altered cardiovascular care flows. During the COVID-19 pandemic, there was a reduction in hospitalizations due to fear of contamination, changes in hospital discharge protocols focused on early discharge, and difficulties in accessing the healthcare system^(28-31)^. These factors may have masked the actual readmission rate, limiting the ability to detect associations between adherence to the MedDiet and clinical outcomes. Studies with larger samples and longer follow-up periods are needed to adequately assess whether adherence to the Mediterranean diet reduces hospital readmissions.

The absence of an association between adherence to the MedDiet and inflammatory biomarkers (CRP) contrasts with studies demonstrating the anti-inflammatory effects of the Mediterranean diet^(13,16,38,39)^. However, a trend toward lower CRP values was observed in adherent patients at the end of follow-up, suggesting a possible benefit that may become significant with longer follow-up. The significant reduction in total cholesterol and LDL cholesterol, regardless of adherence to the MedDiet, may be related to optimized drug therapy during follow-up. Studies such as CORDIOPREV, PREDIMED, and INTERCATH have demonstrated improvements in lipid profile associated with adherence to the MedDiet^(7,13,16)^, a difference that can be attributed to the longer follow-up and larger sample sizes of these clinical trials.

The findings of this study have direct implications for cardiovascular nursing practice, especially in the development of health education and self-care promotion strategies. The identification of factors associated with greater adherence to the MedDiet provides support for personalizing care by developing specific educational approaches that consider age, gender, education, and socioeconomic status. Nursing plays a key role in implementing food education programs during hospitalization, especially for surgical patients, and in establishing post-discharge follow-up protocols to reinforce nutritional guidelines. The integration of nursing into multidisciplinary teams is essential for promoting sustainable behavioral change by leveraging health literacy strategies that account for patients’ individual characteristics and socioeconomic conditions. Nurses, as health educators, have the technical competence and proximity to patients that strategically position them to promote adherence to cardioprotective dietary patterns, contributing to the secondary prevention of cardiovascular diseases and improving the quality of life of individuals with CAD.

From an economic standpoint, promoting adherence to the Mediterranean diet may represent a cost-effective strategy for secondary prevention of CAD, given the potential to reduce readmissions and future cardiovascular events; however, economic analyses are needed to confirm this hypothesis in the Brazilian context.

### Study limitations

The present study has limitations that should be considered when interpreting the results. Not all patients underwent laboratory tests at both time points, limiting the analysis of biomarkers. The collection of data during the COVID-19 pandemic may have influenced the results, as studies have shown changes in food consumption due to socioeconomic inequalities, increased time spent at home, and intensification of disorders such as stress, anxiety, and depression.

Although adapted for Brazilian Portuguese, the MEDAS instrument has not undergone a comprehensive psychometric analysis and requires further validation. The sample profile, restricted to private institutions, limits generalization to populations in the public system. The six-month follow-up may be insufficient to detect significant effects on biomarkers and clinical outcomes. Data extrapolation should be performed with caution.

### Contributions to nursing, health, or public policy

This study offers contributions to cardiovascular nursing practice and public policy. Understanding the factors associated with adherence to the Mediterranean diet provides insights for improving nursing guidance during hospitalization and outpatient follow-up.

Nurses have the competence to plan care and promote self-care through health education focused on cardioprotective eating habits. The findings allow for the personalization of educational interventions considering age, gender, education, and socioeconomic status. The association between surgical treatment and greater adherence highlights hospitalization as an opportunity for structured health education programs.

In the context of public policy, the results support strategies that consider socioeconomic factors, including the availability and access to cardioprotective foods. Nurses can develop educational technologies, follow-up protocols, and health literacy strategies that promote sustainable adherence to healthy eating patterns, contributing to the reduction of cardiovascular morbidity and mortality.

## CONCLUSIONS

Adherence to the Mediterranean diet increased during follow-up, being associated with younger age, female gender, higher education, being economically active, hypertension, and surgical revascularization. There was no association with hospital readmission or inflammatory biomarkers; however, reductions in total and LDL cholesterol were observed.

The findings provide evidence for cardiovascular nursing and highlight the need for personalized educational approaches. The association between surgical treatment and greater adherence suggests that hospitalization represents a valuable opportunity for structured educational interventions. Multicenter studies with larger samples and longer follow-up periods, including populations served by the public health system, are recommended to further investigate the associations among the Mediterranean diet, demographic characteristics, and cardiovascular treatment type.

## Data Availability

The research data are available within the article.
