# Supplementary material for: Factors associated with adherence to the Mediterranean diet in patients with coronary artery disease: a prospective cohort study
Source: Rev Bras Enferm. 2026 Aug 3;79:e20250248. doi: 10.1590/0034-7167-2025-0248 (PMC13432808; doi:10.1590/0034-7167-2025-0248)
Supplement: Translation

# Translation: Fatores associados à adesão à dieta mediterrânea em pacientes com doença arterial coronariana: estudo coorte prospectivo

**DOI:** 10.1590/0034-7167-2025-0248pt

**Authors:** Melissa Alves Cirelli, Juliana de Lima Lopes, Aline Marcadenti, Vinícius Batista Santos, Agueda Maria Ruiz Zimmer Cavalcante, Alba Lucia Bottura Leite de Barros

**Affiliations:** I Universidade Federal de São Paulo. São Paulo, São Paulo, Brasil; II Instituto de Ensino e Pesquisa Hcor. São Paulo, São Paulo, Brasil; III Universidade Federal de Goiás. Goiânia, Goiás, Brasil

**Keywords:** Dieta Mediterrânea, Doença Arterial Coronariana, Fatores Sociodemográficos, Enfermagem, Estudos de Coortes.

## Abstract

**Objetivos:**

verificar a associação entre adesão à dieta mediterrânea (MedDiet), fatores sociodemográficos, clínicos e bioquímicos e a readmissão hospitalar em pacientes com doença arterial coronariana.

**Métodos:**

coorte prospectiva em hospital terciário. A adesão à MedDiet foi analisada na internação e aos três e seis meses após a alta hospitalar pelo *Mediterranean Diet Adherence Screener.* Proteína C-reativa, colesterol total, LDL-colesterol e triglicérides foram verificados na internação e seis meses após a alta; a readmissão foi avaliada aos três e seis meses. A associação foi verificada pelo modelo de equação de estimação generalizada com valores p≤0,05 sendo significativos.

**Resultados:**

participaram 146 pacientes, a maioria masculina, média de 66 anos. A adesão à MedDiet aumentou de 3% para 41% e 47% no seguimento e associou-se à idade, sexo feminino, ensino superior completo, atividade econômica e revascularização miocárdica prévia.

**Conclusões:**

a adesão à MedDiet esteve associada a fatores sociodemográficos e clínicos.

## INTRODUÇÃO

As Doenças Cardiovasculares (DCVs) constituem um problema de saúde pública global, cuja prevalência crescente gera impacto financeiro e compromete a produtividade e qualidade de vida dos indivíduos^(1)^. Segundo dados atualizados da American Heart Association (2025), as DCVs permanecem como a principal causa de mortalidade mundial, com tendência de crescimento em países em desenvolvimento^(1)^.

No Brasil, as DCVs representam a principal causa de morte, sendo a Doença Arterial Coronariana (DAC) responsável pela maior proporção dos óbitos cardiovasculares, seguida pelo acidente vascular cerebral. O impacto econômico é considerável, com custos anuais do Sistema Único de Saúde (SUS) superiores a R$ 1 bilhão em procedimentos cardiovasculares^(2)^. A mortalidade por DAC apresenta associação inversa com o nível socioeconômico, característica marcante dos países em desenvolvimento, agravada pela presença de fatores de risco modificáveis como obesidade, tabagismo, sedentarismo e padrões alimentares inadequados^(2)^.

A fisiopatologia da DAC envolve complexos mecanismos aterogênicos, nos quais fatores de risco, como dislipidemia (DLP), hipertensão arterial sistêmica (HAS) e tabagismo, desempenham papel fundamental na progressão da doença cardiovascular. Esses fatores promovem disfunção endotelial e aumentam a permeabilidade da camada íntima às lipoproteínas plasmáticas, especialmente as de baixa densidade (LDL)^(3)^. Esse processo desencadeia resposta inflamatória crônica que pode resultar em instabilidade da placa aterosclerótica, ruptura com ativação da cascata de coagulação e, consequentemente, oclusão arterial aguda^(4)^.

Nesse contexto, a dieta mediterrânea (MedDiet)^(3)^ tem emergido como estratégia nutricional de destaque, sendo amplamente recomendada por diretrizes e consensos internacionais para prevenção primária e secundária das DCVs^(4-6)^. Estudos demonstram que a MedDiet exerce efeitos anti-inflamatórios e antioxidantes evidenciados pela redução de biomarcadores inflamatórios e melhora da função endotelial, conferindo proteção cardiovascular significativa. Ensaios clínicos randomizados de grande impacto, como o CORDIOPREV^(7)^ e o Lyon Heart Study^(8)^, comprovaram a efetividade da MedDiet na prevenção secundária das DCVs, demonstrando redução substancial de eventos cardiovasculares maiores.

Entretanto, diversos fatores sociodemográficos, clínicos e bioquímicos podem influenciar na adesão à MedDiet, especialmente em populações não mediterrâneas. Embora a literatura internacional seja robusta quanto aos benefícios da MedDiet, estudos que avaliem os determinantes da adesão a esse padrão alimentar, no contexto da prevenção secundária cardiovascular em países latino-americanos, permanecem escassos. Essa lacuna é particularmente relevante considerando-se as diferenças culturais, socioeconômicas e de disponibilidade alimentar que podem impactar a implementação e manutenção desse padrão dietético em populações brasileiras com DAC estabelecida.

## OBJETIVOS

Identificar a associação entre a adesão à MedDiet e fatores sociodemográficos, clínicos, bioquímicos e a readmissão hospitalar em pacientes com doença arterial coronariana.

## MÉTODOS

### Aspectos éticos

O estudo foi submetido e aprovado pelos comitês de ética em pesquisa das instituições envolvidas, em conformidade com a Resolução nº 466/2012 do Conselho Nacional de Saúde.

### Desenho do estudo

Trata-se de estudo de coorte prospectivo, norteado pela ferramenta STROBE^(9)^, realizado em um hospital geral privado, terciário, de grande porte, situado em São Paulo. O estudo foi conduzido em duas unidades assistenciais: Unidade Crítica Cardiológica (UCC), com 45 leitos de cuidados semicríticos, média mensal de 56 internação por DAC, tempo médio de permanência de três dias e taxa de ocupação de 95%; e Unidade de Internação (UI), com 31 leitos de baixa complexidade para afecções circulatórias, tempo médio de permanência de três dias e taxa de ocupação de 81%.

### Cálculo amostral e período de coleta

A coleta de dados ocorreu entre julho de 2021 e julho de 2022. O cálculo amostral baseou-se em estudo prévio^(10)^ que identificou prevalência de 25,4% de adesão à dieta mediterrânea em população de 18.991 indivíduos com idade superior a 35 anos. Considerando amostra infinita, intervalo de confiança de 80% e erro amostral de 5%, obteve-se tamanho mínimo de 125 pacientes. Prevendo-se perda de 20%, estabeleceu-se amostra de 149 pacientes, resultando em amostra final de 146 pacientes com diagnóstico de DAC.

A fórmula utilizada foi: n = [EDFF*Np(1-p)]/ [(d2/Z21-α/2*(N-1)+p*(1-p)].

### Critérios de elegibilidade

Foram incluídos pacientes maiores de 18 anos internados por doença arterial coronariana (DAC), classificada conforme diretrizes clínicas estabelecidas:

1. DAC aguda: definida pela presença de síndrome coronariana aguda, diagnosticada mediante critérios clínicos, eletrocardiográficos e laboratoriais, incluindo: - angina instável (dor torácica em repouso com alterações eletrocardiográficas transitórias); - infarto agudo do miocárdio com supradesnivelamento do segmento ST (elevação ≥1mm em duas derivações contíguas); - infarto agudo do miocárdio sem supradesnivelamento do segmento ST (depressão do segmento ST ≥0,5mm e/ou inversão da onda T, associada à elevação de biomarcadores cardíacos).
2. DAC Crônica: caracterizada por doença coronariana estável diagnosticada por métodos não invasivos (teste ergométrico, ecocardiograma de estresse, cintilografia miocárdica) ou invasivos (cineangiocoronariografia), incluindo: - angina estável (dor torácica aos esforços, com padrão previsível); - dispneia de esforço relacionada à isquemia miocárdica; - insuficiência cardíaca de etiologia isquêmica ou disfunção ventricular esquerda; - pacientes assintomáticos ou com sintomas estáveis há menos de um ano, após síndrome coronariana aguda; - pacientes submetidos à revascularização miocárdica (percutânea ou cirúrgica) há menos de um ano; - pacientes com sintomas estáveis há mais de um ano, após primeiro diagnóstico ou revascularização; - suspeita de doença vasoespástica ou microvascular com prova funcional positiva. 3. DAC detectada em rastreamento de pacientes assintomáticos.

Foram excluídos os seguintes casos:

1. Pacientes oncológicos em quimioterapia ativa: devido à necessidade de prescrição dietética específica para essa população que poderia interferir na avaliação da adesão à dieta mediterrânea.
2. Comprometimento cognitivo ou neurológico: pacientes com confusão mental, rebaixamento do nível de consciência ou sob sedação avaliados diariamente pelas equipes médica e de enfermagem mediante: - Escala de Coma de Glasgow <15 pontos; - avaliação clínica documentada em prontuário quanto ao estado de consciência; - uso de sedativos que comprometessem a capacidade de resposta.
3. Alergias alimentares: indivíduos com alergias, documentadas em prontuário, a componentes da dieta mediterrânea (oleaginosas, peixes, azeite de oliva, entre outros), conforme registro médico.
4. Impossibilidade de contato: pacientes sem resposta ao contato telefônico após três tentativas, em horários distintos, foram considerados como perda amostral, não como critério de exclusão, conforme recomendações do *checklist* STROBE para estudos observacionais.

### Protocolo e período do estudo

Recrutamento: identificação diária pela pesquisadora por busca ativa de pacientes com DAC e programação de alta hospitalar. Os pacientes eram abordados presencialmente na unidade de internação e convidados a participar mediante explicação dos procedimentos do estudo.

Instrumento de coleta: aplicação de questionário estruturado abrangendo variáveis sociodemográficas e clínicas selecionadas mediante revisão narrativa, nas bases de dados Medline, Lilacs e Cochrane (artigos dos últimos cinco anos, português/inglês/espanhol). As informações foram obtidas diretamente com o paciente e complementadas por dados dos prontuários.

Variáveis coletadas: sociodemográficas - sexo, idade, raça, estado civil, escolaridade, ocupação; clínicas - tempo de internação, tipo de DAC, índice de massa corpórea (IMC), comorbidades (depressão, hipertensão, diabetes, dislipidemia); e laboratoriais - colesterol total, LDL-colesterol, triglicerídeos e proteína C-reativa (PCR)^(11-14)^. O tempo médio de entrevista foi de 30 minutos por participante.

O desfecho primário foi analisado pelo *Mediterranean Diet Adherence Screener* (MEDAS), instrumento traduzido e adaptado para o português brasileiro^(15)^, composto por 14 questões com escore de 0-1 cada. Uma pontuação ≥10 indica maior adesão à MedDiet. O instrumento foi aplicado em três momentos: presencialmente, até 48 horas antes da alta hospitalar; e em seguimento por contato telefônico aos três e aos seis meses após a alta hospitalar.

Os desfechos secundários foram analisados pelos valores de PCR, colesterol total, LDL-colesterol e triglicérides obtidos durante a internação e após seis meses da alta hospitalar, por meio dos dados contidos no prontuário e/ou por contato telefônico. A coleta de biomarcadores ocorreu durante a internação, sendo realizada pela equipe de enfermagem conforme os seguintes protocolos institucionais: garroteamento 7,5-10cm do local de punção, antissepsia com álcool 70% em movimentos espirais, punção venosa com ângulo 30º-45º e armazenamento em tubos específicos para análise no laboratório Fleury®.

Os valores de colesterol, triglicérides e LDL-colesterol estão em conformidade com a atualização da Diretriz Brasileira de Dislipidemias e Prevenção da Aterosclerose - 2017, da Sociedade Brasileira de Cardiologia^(3)^. A PCR foi determinada pelo método de imunoturbidimetria e os valores de referência para classificar o risco cardiovascular são: abaixo de 0,1 mg/dL: risco baixo; de 0,1 a 0,3 mg/dL: risco intermediário; e acima de 0,3 mg/dL: risco aumentado. Quanto ao exame de colesterol total, o valor de referência desejável, com ou sem jejum de 12 horas, idade maior ou igual a 20 anos, é menor que 190 mg/dL. Quanto aos triglicérides, o valor de referência desejável para idade acima de 20 anos, com jejum de 12 horas, é menor que 150 mg/dL. E quanto ao LDL-colesterol, o valor de referência para idade acima de 20 anos, com ou sem jejum de 12 horas, é estabelecido para avaliação primária do perfil lipídico como ótimo: menor de 100 mg/dL; desejável: de 100 a 129 mg/dL; limítrofe: de 130 a 159 mg/dL; alto: de 160 a 189 mg/dL; muito alto: maior ou igual a 190 mg/dL. A readmissão hospitalar foi detectada por meio do relato do participante, durante contato telefônico, após três e seis meses da alta hospitalar.

### Análises dos resultados

As variáveis qualitativas foram descritas por frequência absoluta e relativa; as quantitativas, por médias de tendência central e dispersão. A adesão ao MEDAS foi estimada com intervalo de confiança de 95%. Modelos de Equação de Estimação Generalizada (*GEE - Generalized Estimating Equation*) avaliaram fatores associados à adesão, estimando risco relativo mediante modelo múltiplo.

Para os dados faltantes >5%, utilizou-se imputação múltipla (MICE - *Multiple Imputation With Chained Equations).* Variáveis com mais de 50% de dados ausentes foram excluídas das análises. Teste de Wilcoxon comparou distribuições de variáveis quantitativas. Modelos Lineares Generalizados Mistos (MLGM) avaliaram evolução dos biomarcadores com teste da razão de verossimilhanças para termos de interação. O nível de significância adotado foi de p<0,05.

## RESULTADOS

Foram rastreados 334 pacientes com DAC durante o período de coleta. Destes, 181 atenderam aos critérios de elegibilidade e aceitaram participar do estudo mediante assinatura do TCLE. Durante o seguimento, ocorreram as seguintes perdas: 33 pacientes não responderam a nenhum contato telefônico (terceiro e sexto mês); 18 não responderam especificamente ao MEDAS no terceiro mês; dois não responderam ao contato do sexto mês; e dois solicitaram retirada do estudo. A amostra final analisada foi composta por 146 pacientes que responderam a pelo menos um contato telefônico, sendo que 126 participantes completaram todo o seguimento (terceiro e sexto mês), conforme apresentado na Figura 1.

**Figura 1:** Fluxograma de seleção e seguimento dos participantes conforme diretrizes STROBE, São Paulo, São Paulo, Brasil, 2021-2022

A Tabela 1 apresenta a caracterização sociodemográfica e clínica dos 146 participantes. Em relação aos biomarcadores, a maioria dos pacientes apresentou PCR indicativo de alto risco cardiovascular (>0,3 md/dL), seguido de risco intermediário (0,1-0,3 mg/dL). Treze pacientes não realizaram coleta de PCR durante a internação. Quanto ao perfil lipídico, entre os pacientes que realizaram coleta durante a internação, a maioria apresentou valores de colesterol total e triglicerídeos dentro da faixa classificada como desejável segundo diretrizes brasileiras.

**Tabela 1:** Caracterização sociodemográfica e clínica e associação com a adesão à dieta do mediterrâneo, ao longo do tempo, de pacientes com Doença Arterial Coronariana (N=146), São Paulo, São Paulo, Brasil

Variável	N = 146^*^	RR†	IC95%	Valor de *p*
Tempo de internação (dias)				
Média (DP)	6,70 (11,56)	0,992	0,976-1,008	0,321
Idade				
Média (DP)	66,27 (12,09)	0,984	0,969-0,999	0,042
Sexo				
Masculino	117 (80,14%)	0,566	0,350-0,917	0,021
Feminino	29 (19,86%)	Referência		
Escolaridade				
Superior	126 (86,30%)	Referência		
Ensino Médio completo	16 (10,96%)	0,939	0,484-1,825	0,854
Ensino Fundamental	4 (2,74%)	0,410	0,183-0,917	0,030
Estado civil				
Mora com companheiro(a)	131 (89,73%)			
Sem companheiro(a)	15 (10,27%)	0,835	0,398-1,753	0,634
Raça				
Branca	143 (97,95%)			
Não branca	3 (2,05%)			
Classificação da ocupação				
Economicamente ativo	123 (84,25%)	Referência		
Não economicamente ativo	23 (15,75%)	0,377	0,149-0,953	0,039
Diagnóstico médico				
DAC crônica	93 (63,70%)	1,236	0,810-1,888	0,326
DAC aguda	53 (36,30%)			
Antecedentes pessoais				
Sedentarismo	111 (76,03%)	Referência		
Hipercolesterolemia	110 (75,34%)	1,037	0,637-1,689	0,883
Hipertensão Arterial Sistêmica	102 (69,86%)	1,754	1,155-2,662	0,008
Tratamento realizado				
Angioplastia	115 (78,77%)	Referência		
Clínico	18 (12,33%)	0,935	0,507-1,727	0,831
Cirúrgico	13 (8,90%)	1,614	1,018-2,558	0,042
IMC				
Média (DP)	27,51 (3,97)	0,985	0,942-1,031	0,517
Máximo/Mínimo	17,48, 42,39			
Classificação IMC				
Sobrepeso	81 (55,48%)			
Normal	33 (22,60%)			
Obeso	31 (21,23%)			
Baixo peso	1 (0,68%)			
PCR				
Média (DP)	0,65 (1,18)			
Máximo/Mínimo	0,03, 7,67			
Ausência de resultado	13			
Indicador de Risco Cardiovascular de acordo com PCR				
Alto (acima de 0,3 mg/dL)	52 (39,10%)			
Baixo (abaixo 0,1 mg/dL)	35 (26,32%)			
Intermediário (0,1 mg/dL a 0,3 mg/dL)	46 (34,59%)			
Ausência de resultado	13			
Colesterol total				
Média (DP)	167,09 (51,94)			
Colesterol desejável < 190				
Sim	72 (71,29%)			
LDL				
Média (DP)	96,36 (42,80)			
LDL desejável <100				
Sim	64 (63,37%)			
Triglicerídeos				
Média (DP)	152,87 (106,01)			
Triglicerídeos - desejável < 150				
Sim	68 (67,33%)			
Medicamentos prescritos				
Antiagregantes	113 (77,40%)	1,053	0,685-1,618	0,814
Hipolipomiantes	103 (70,55%)			
Betabloqueadores	56 (38,36%)	1,077	0,698-1,660	0,738

IRR† - Incidência de Risco Relativo; ^^*^^N (%); DP - Desvio Padrão; IC - Intervalo de Confiança; IMC - Índice de Massa Corpórea; DAC - Doença Arterial Coronariana; PCR - Proteína C-reativa; HAS - Hipertensão Arterial Sistêmica; DM - Diabetes Mellitus.

Na análise bivariada, as variáveis que apresentaram associação estatisticamente significativa com maior adesão ao MEDAS foram: menor idade, sexo feminino, ensino superior, ser economicamente ativo, presença de hipertensão arterial e ter sido submetido a tratamento cirúrgico para DAC.

A Figura 2 demonstra a proporção de participantes aderentes à MedDiet (MEDAS ≥10) nos três momentos de avaliação. Observou-se aumento progressivo da adesão ao longo do seguimento: internação (*baseline*), três meses e seis meses pós-alta hospitalar. Esse achado indica melhoria na aderência ao padrão alimentar mediterrâneo durante o período de acompanhamento.

**Figura 2:** Proporção da adesão à dieta do mediterrâneo durante o seguimento de pacientes com Doença Arterial Coronariana N=146, São Paulo, São Paulo, Brasil

A Tabela 2 apresenta a relação entre a adesão à MedDiet e a readmissão hospitalar nos períodos de três e seis meses pós-alta. Não foi identificada diferença estatisticamente significativa entre os grupos com maior e menor adesão à dieta quanto às taxas de readmissão (p>0,05). As taxas de readmissão mantiveram-se baixas durante todo o período de seguimento.

**Tabela 2:** Relação da adesão à dieta mediterrânea da internação e a readmissão hospitalar após três e seis meses da alta hospitalar, São Paulo, São Paulo, Brasil, 2025

Variável	Readmissão hospitalar (T0)			Valor de *p* ^§^
	Total N = 146‡	Não n = 143‡	Sim n = 3‡	
**Adesão ao MEDAS T0**				0.101
Não	141 (96,55%)	139 (97,18%)	2 (66,67%)	
Sim	5 (3,45%)	4 (2,82%)	1 (33,33%)	
	**Readmissão hospitalar (T6)**			
	**Total N = 144‡**	**Não n = 135‡**	**Sim n = 9‡**	
**Adesão ao MEDAS T3**				0,145
Não	76 (60,32%)	69 (58,47%)	7 (87,50%)	
Sim	50 (39,68%)	49 (41,53%)	1 (12,50%)	
Ausência de resultado	18	17	1	

‡*n (%); §Fisher’s exact test; MEDAS - Mediterranean Diet Adherence Screener; T0 - tempo na internação; T6 - tempo após seis meses da alta hospitalar.*

Conforme apresentado na Tabela 3, a adesão à MedDiet não demonstrou associação estatisticamente significativa com os valores de biomarcadores aos seis meses de seguimento. Entretanto, observou-se redução significativa dos níveis de colesterol total e LDL-colesterol, ao longo do tempo, em toda a amostra (p<0,05), independentemente do grau de adesão à dieta mediterrânea. A análise não identificou influência da interação entre adesão dietética e uso de medicamentos hipolipemiantes na evolução dos biomarcadores.

**Tabela 3:** Associação do valor de PCR, colesterol total, LDL-colesterol e triglicerídeos com adesão à dieta do mediterrâneo no seguimento de seis meses de pacientes com Doença Arterial Coronariana, São Paulo, São Paulo, Brasil

Covariável	GLMM (*Generalized Linear Mixed Model*)											
	Colesterol total			Triglicerídeos			LDL-colesterol			PCR		
	Beta	IC 95%	Valor de *p*	Beta	IC 95%	Valor de *p*	Beta	IC 95%	Valor de *p*	Beta	IC 95%	Valor de *p*
**Tempo**												
T0	-	-	-	-	-	-	-	-	-	-	-	-
T6	-32,49	-46,5 -18,3	<0,001	0,812	0,69 - 0,95	0,012	0,76	0,64 - 0,89	<0,001	0,75	-0,09 - 1,60	0,082
**Adesão ao MEDAS**												
Não	-	-	-	-	-	-	-	-	-	-	-	-
Sim	-1,86	-21,3 - 1,02	0,841	0,850	0,67 - 1,06	0,162	0,95	0,75 - 1,20	0,701	0,49	-0,69 - 1,68	0,413
**Hipolipomiante**												
Não	-	-	-	-	-	-	-	-	-	-	-	-
Sim	4,32	-34,07 -25,38	0,776	0,901	0,62 - 1,30	0,578	0,896	0,62 - 1,29	0,558	0,34	-0,93 - 1,63	0,594

*GLMM - Generalized Linear Mixed Model; MEDAS - Mediterranean Diet Adherence Screener; T0T6 - tempo internação e após seis meses de alta hospitalar; M0 - MEDAS internação; M6 - MEDAS seis meses.*

## DISCUSSÃO

Os resultados demonstram que a adesão à dieta mediterrânea aumentou progressivamente ao longo do seguimento de seis meses e esteve associada a menor idade, sexo feminino, ensino superior, ser economicamente ativo, HAS e revascularização do miocárdio. Não foi observada relação entre adesão à MedDiet e readmissão hospitalar ou biomarcadores inflamatórios; entretanto, verificou-se redução significativa do colesterol total e LDL-colesterol durante o período de acompanhamento.

A caracterização sociodemográfica do presente estudo é consistente com outras coortes cardiovasculares quanto à predominância do sexo masculino, da média de idade e da raça branca^(16-19)^. A maior prevalência masculina reflete dados epidemiológicos da DAC^(1-3,19-21)^ associada à menor adesão dos homens aos cuidados preventivos e à busca tardia por serviços de saúde^(21)^. A associação entre sexo feminino e maior adesão à MedDiet corrobora achados internacionais^(22-26)^, possivelmente relacionados ao fato de que mulheres tendem a adotar comportamentos alimentares mais saudáveis, consumir maior quantidade de frutas e vegetais e apresentar menor consumo de álcool comparativamente aos homens^(22,26,27)^.

Contrariando tendências descritas na literatura nacional e internacional que associam maior adesão à MedDiet a faixas etárias mais avançadas^(16,22,25,27)^, este estudo identificou que indivíduos mais jovens apresentaram maior aderência ao padrão alimentar mediterrâneo. Esse resultado pode estar relacionado ao impacto da hospitalização em pacientes mais jovens, que podem apresentar maior motivação para mudanças comportamentais após evento cardiovascular agudo, incluindo adoção de hábitos alimentares cardioprotetores, cessação do tabagismo e início de atividade física regular. Adicionalmente, o contexto pandêmico pode ter influenciado esse achado, uma vez que estudos multicêntricos realizados durante a pandemia de COVID-19 demonstraram mudanças significativas nos padrões alimentares, com aumento do consumo de frutas e hortaliças entre jovens adultos, especialmente no Brasil^(28-31)^. A maior consciência sobre a saúde e imunidade durante esse período, associada ao maior acesso à informação sobre nutrição por meio de plataformas digitais, pode ter contribuído para maior adesão a padrões alimentares saudáveis nessa faixa etária.

A associação entre maior escolaridade, ser economicamente ativo e maior adesão à MedDiet confirma achados da literatura^(25,32)^. O nível socioeconômico constitui determinante fundamental no acesso^(2)^ a comportamentos saudáveis e escolhas alimentares adequadas^(22,33)^. A escolaridade influencia diretamente no controle de fatores de risco cardiovascular e na adesão aos hábitos saudáveis, uma vez que o aumento do índice socioeconômico está associado à maior consciência sobre saúde e melhores condições para aquisição de alimentos de qualidade^(2,19,21,23,32-35)^. No contexto brasileiro, as desigualdades são agravadas por instabilidades políticas e econômicas que promovem disparidades no acesso à alimentação adequada, em termos de qualidade e quantidade^(28-31)^.

A associação significativa entre a presença de HAS e a maior adesão à MedDiet pode estar relacionada ao acompanhamento profissional especializado e maior acesso à assistência à saúde, fatores que favorecem modificações comportamentais. Estudo prévio demonstrou maior adesão à MedDiet em indivíduos com doenças crônicas estabelecidas, como HAS, DM e DLP^(36)^, possivelmente em virtude da maior consciência sobre a importância da alimentação no controle dessas condições.

A associação observada entre adesão à MedDiet e a revascularização cirúrgica constitui achado inovador que merece investigação aprofundada. Embora a angioplastia seja o procedimento de escolha (58,9%) comparativamente à revascularização cirúrgica (20,1%)^(2)^, pacientes submetidos a cirurgia cardíaca vivenciam experiência hospitalar mais complexa e prolongada. Essa associação pode ser explicada pelo impacto psicológico da cirurgia cardíaca, que representa marco significativo na percepção de gravidade da doença, promovendo maior motivação para mudanças comportamentais^(37)^. Adicionalmente, pacientes cirúrgicos recebem orientações mais estruturadas sobre modificações do estilo de vida durante período de internação prolongado, e o processo de reabilitação pós-cirúrgica envolve equipe multidisciplinar, incluindo profissionais de enfermagem especializados em educação nutricional.

O baixo número de readmissão observado deve ser interpretado considerando-se o contexto pandêmico, que alterou os fluxos assistenciais cardiovasculares. Durante a pandemia da COVID-19, observou-se redução de internações por receio de contaminação, modificação nos protocolos de alta hospitalar com foco na desospitalização precoce e dificuldades de acesso ao sistema de saúde^(28-31)^. Esses fatores podem ter mascarado a taxa real de readmissões, limitando a capacidade de detectar associações entre adesão à MedDiet e os desfechos clínicos. Estudos com amostras maiores e períodos de seguimento mais prolongado são necessários para investigar adequadamente se a adesão à dieta mediterrânea minimiza eventos de readmissão hospitalar.

A ausência de associação entre adesão à MedDiet e biomarcadores inflamatórios (PCR) contrasta com estudos que demonstram efeitos anti-inflamatórios da dieta mediterrânea^(13,16,38,39)^. Entretanto, observou-se tendência de menores valores de PCR em pacientes aderentes ao final do seguimento, sugerindo possível benefício que pode tornar-se significativo com maior tempo de acompanhamento. A redução significativa de colesterol total e LDL-colesterol, independentemente da adesão à MedDiet, pode estar relacionada à otimização da terapia medicamentosa durante o seguimento. Estudos como o CORDIOPREV, PREDIMED, INTERCATH demonstraram melhora no perfil lipídico especificamente associada à adesão à MedDiet^(7,13,16)^, diferença que pode ser atribuída ao maior tempo de seguimento e tamanho amostral desses ensaios clínicos.

Os achados deste estudo têm implicações diretas para a prática da enfermagem cardiovascular, especialmente no desenvolvimento de estratégias de educação em saúde e promoção do autocuidado. A identificação de fatores associados à maior adesão à MedDiet fornece subsídios para a personalização do cuidado por meio do desenvolvimento de abordagens educativas específicas considerando idade, sexo, escolaridade e condição socioeconômica. A enfermagem desempenha papel fundamental na implementação de programas de educação alimentar durante a hospitalização, especialmente para pacientes cirúrgicos, e no estabelecimento de protocolos de acompanhamento pós-alta para reforço das orientações nutricionais. A integração da enfermagem em equipes multidisciplinares é essencial para a promoção de mudanças comportamentais sustentáveis, utilizando estratégias de letramento em saúde que considerem as características individuais dos pacientes e suas condições socioeconômicas. O enfermeiro, como educador em saúde, possui competência técnica e proximidade com o paciente, o que o posiciona estrategicamente para promover a adesão a padrões alimentares cardioprotetores, contribuindo para a prevenção secundária das doenças cardiovasculares e melhoria da qualidade de vida dos indivíduos com DAC.

Do ponto de vista econômico, a promoção da adesão à dieta mediterrânea pode representar estratégia custo-efetiva na prevenção secundária da DAC, considerando o potencial de redução de readmissões e eventos cardiovasculares futuros, embora estudos de análise econômica sejam necessários para confirmar essa hipótese no contexto brasileiro.

### Limitações do estudo

O estudo apresenta limitações que devem ser consideradas na interpretação dos resultados. Nem todos os pacientes realizaram a coleta dos exames laboratoriais nos dois momentos avaliados, limitando a análise dos biomarcadores. A coleta durante a pandemia da COVID-19 pode ter influenciado os resultados, período em que estudos evidenciaram mudanças no consumo alimentar em razão das desigualdades socioeconômicas, da maior permanência domiciliar e intensificação de transtornos como estresse, ansiedade e depressão.

O instrumento MEDAS, embora adaptado para o português brasileiro, não passou por análises psicométricas completas, necessitando validação definitiva. O perfil da amostra, restrito a instituição privada, limita a generalização para populações do sistema público. O seguimento de seis meses pode ser insuficiente para detectar efeitos significativos sobre biomarcadores e desfechos clínicos. A extrapolação dos dados deve ser realizada com cautela.

### Contribuições para a enfermagem, saúde ou política pública

Este estudo oferece contribuições para a prática da enfermagem cardiovascular e políticas públicas. A compreensão dos fatores associados à adesão à dieta mediterrânea fornece subsídios para aprimoramento das orientações de enfermagem durante a hospitalização e o seguimento ambulatorial.

O enfermeiro possui competência para planejar assistência e promover autocuidado através de educação em saúde direcionada a hábitos alimentares cardioprotetores. Os achados permitem a personalização das intervenções educativas considerando idade, sexo, escolaridade e condição socioeconômica. A associação entre tratamento cirúrgico e maior adesão destaca a hospitalização como oportunidade para programas estruturados de educação em saúde.

No contexto das políticas públicas, os resultados subsidiam estratégias que consideram recursos socioeconômicos, disponibilidade e acesso a alimentos cardioprotetores. A enfermagem pode desenvolver tecnologias educativas, protocolos de seguimento e estratégias de letramento em saúde que favoreçam a adesão sustentável a padrões alimentares saudáveis, contribuindo para a redução da morbimortalidade cardiovascular.

## CONCLUSÕES

A adesão à dieta mediterrânea aumentou durante o seguimento, associando-se a menor idade, sexo feminino, ensino superior, ser economicamente ativo, hipertensão arterial e revascularização cirúrgica. Não houve associação com readmissão hospitalar ou biomarcadores inflamatórios; entretanto, observou-se redução do colesterol total e LDL-colesterol.

Os achados fornecem evidências para a enfermagem cardiovascular, destacando a necessidade de abordagens educativas personalizadas. A associação entre tratamento cirúrgico e maior adesão sugere que a hospitalização representa oportunidade valiosa para intervenções educativas estruturadas. Recomenda-se a realização de estudos multicêntricos com amostras maiores e períodos de seguimento mais prolongados, incluindo populações atendidas no sistema público de saúde, para investigar mais profundamente as associações entre dieta mediterrânea, características demográficas e tipo de tratamento cardiovascular.

## Data Availability

Os dados de pesquisa estão disponíveis no corpo do artigo.
